# Machine Learning-Assisted Screening of Herbal Medicine Extracts as Vaccine Adjuvants

**DOI:** 10.3389/fimmu.2022.847616

**Published:** 2022-05-19

**Authors:** Kou Hioki, Tomoya Hayashi, Yayoi Natsume-Kitatani, Kouji Kobiyama, Burcu Temizoz, Hideo Negishi, Hitomi Kawakami, Hiroyuki Fuchino, Etsushi Kuroda, Cevayir Coban, Nobuo Kawahara, Ken J. Ishii

**Affiliations:** ^1^Division of Vaccine Science, Department of Microbiology and Immunology, International Vaccine Design Center (vDesC), The Institute of Medical Science, The University of Tokyo (IMSUT), Tokyo, Japan; ^2^Laboratory of Mockup Vaccine, Center for Vaccine and Adjuvant Research Center (CVAR), National Institutes of Biomedical Innovation, Health and Nutrition (NIBIOHN), Osaka, Japan; ^3^Laboratory of Bioinformatics, Artificial Intelligence Center for Health and Biomedical Research, National Institutes of Biomedical Innovation, Health and Nutrition (NIBIOHN), Osaka, Japan; ^4^Research Center for Medicinal Plant Resources, National Institutes of Biomedical Innovation, Health and Nutrition (NIBIOHN), Ibaraki, Japan; ^5^Department of Immunology, Hyogo College of Medicine, Hyogo, Japan; ^6^Division of Malaria Immunology, Department of Microbiology and Immunology, International Vaccine Design Center (vDesC), The Institute of Medical Science, The University of Tokyo (IMSUT), Tokyo, Japan; ^7^Immunology Frontier Research Center (IFReC), Osaka University, Osaka, Japan

**Keywords:** vaccine, adjuvant, machine learning, herbal extracts, mouse, human

## Abstract

Adjuvants are important vaccine components, composed of a variety of chemical and biological materials that enhance the vaccine antigen-specific immune responses by stimulating the innate immune cells in both direct and indirect manners to produce a variety cytokines, chemokines, and growth factors. It has been developed by empirical methods for decades and considered difficult to choose a single screening method for an ideal vaccine adjuvant, due to their diverse biochemical characteristics, complex mechanisms of, and species specificity for their adjuvanticity. We therefore established a robust adjuvant screening strategy by combining multiparametric analysis of adjuvanticity *in vivo* and immunological profiles *in vitro* (such as cytokines, chemokines, and growth factor secretion) of various library compounds derived from hot-water extracts of herbal medicines, together with their diverse distribution of nano-sized physical particle properties with a machine learning algorithm. By combining multiparametric analysis with a machine learning algorithm such as rCCA, sparse-PLS, and DIABLO, we identified that human G-CSF and mouse RANTES, produced upon adjuvant stimulation *in vitro*, are the most robust biological parameters that can predict the adjuvanticity of various library compounds. Notably, we revealed a certain nano-sized particle population that functioned as an independent negative parameter to adjuvanticity. Finally, we proved that the two-step strategy pairing the negative and positive parameters significantly improved the efficacy of screening and a screening strategy applying principal component analysis using the identified parameters. These novel parameters we identified for adjuvant screening by machine learning with multiple biological and physical parameters may provide new insights into the future development of effective and safe adjuvants for human use.

## Introduction

Vaccines are the most successful preventive medicine and their importance has been further emphasized during the COVID-19 pandemic (1–3). Considering the fact that there are still numerous infectious diseases for which vaccines have not yet been developed (1, 4, 5), further development of vaccines remains an important issue (6). Moreover, it is important to prepare for the next pandemic which could possibly occur in the future. For the efficacy of vaccines, adjuvant is one of the most important components that control the type and magnitude of immune response (1, 6–9). Therefore, adjuvant responses determine the quality and application of the vaccine by the induction of optimal immune response against pathogens (6, 7, 9, 10); however, there are only a limited number of clinically approved adjuvants such as aluminum salts (alum), Monophosphoryl Lipid A (MPLA), and MF59 (6, 7).

Due to the lack of clinically available adjuvants, the development of novel adjuvants is in high demand to fight against multiple diseases (6). It has been proven that many types of materials, such as nucleic acids, lipids, and polysaccharides, possess adjuvant properties (6, 11–14). However, a substantial portion of them, particularly clinically used adjuvants such as alum, have been empirically developed. Therefore, there is a considerable methodological requirement to develop new adjuvants. Although the factors involved in activating immune responses have been well studied (15, 16), *in vitro* parameters that reflect the adjuvanticity *in vivo* of adjuvants remain unclear.

Previous adjuvant studies commonly examined cytokine production from immune cells activated by adjuvants to evaluate their activity *in vitro* (17–20) because the effect of adjuvants is mainly mediated by the activation of innate immune cells such monocytes, macrophages, neutrophils, dendritic cells, and even no-immune cells, leading to the induction of various cytokines, such as pro-inflammatory cytokines. However, cytokines that most reflect adjuvanticity *in vivo* remain unclear; therefore, it is necessary to reevaluate adjuvant-induced cytokine response profiles as the sole biological properties of adjuvants. On the other hand, the physical property of adjuvants, such as the particle formation or size, are found to be important factors for the *in vivo* adjuvant effect (14, 21–28). Although this knowledge has been limited, determination of the physical properties of adjuvants may have a better probability to reflect adjuvanticity *in vivo*.

Natural compounds such as plant-derived compounds used in traditional Chinese herbal medicines have long been considered as seeds for new drugs (29–34). In particular, the biocompatibility of herbal medicine-derived compounds is expected to be high because they have already been registered as drugs for multiple applications in humans. Certain herbal medicine extracts have also been known to activate innate immune responses and act as adjuvants (35–63). Therefore, herbal medicines are advantageous in the discovery of novel adjuvants. We utilized herbal medicine extracts applied as Kampo medicines in Japan as the library for this study. Recently, it has been reported that nanoparticles contained in boiling herbal water extracts exhibited an immunostimulant effect *in vitro* (64). Thus, we predicted that herbal medicine extracts would have diverse biological and physical properties, leading to adjuvanticity *in vivo*.

Here, we aimed to predict the parameters important for adjuvanticity *in vivo* of unknown compounds together with their cytokine response profiles and physical properties. To achieve this, by comparing with seven known water-soluble adjuvants as positive controls for validation, we comprehensively examined 73 types of hot water extracts of herbal medicines deposited as library compounds and obtained their biological and physical properties in humans and mice. For analysis, we utilized three different machine learning algorithms, regularized canonical correlation analysis (rCCA), sparse partial least squares (sparse-PLS), and data integration analysis for biomarker discovery using the latent component method for omics studies (DIABLO).

We identified that nano-sized particles in herbal medicine extracts and soluble adjuvants such as TLR and STING ligand were detectable by flow cytometry and that the distributions based on their size and density were extremely diverse. Based on the machine learning analyses of our dataset, we found that human G-CSF (hG-CSF) and mouse RANTES (mRANTES) are the best positive parameters, and the particle proportion of small particle population (a FSC^low^ SSC^low^ population) is the best negative parameter that reflects the adjuvanticity *in vivo* of soluble adjuvants.

## Materials and Methods

### Mice

Female C57BL/6 mice (6–8 weeks old) were purchased from CLEA Japan, Inc. (Tokyo, Japan). All mouse experiments were performed according to the appropriate laws and guidelines approved by the National Institutes of Biomedical Innovation, Health and Nutrition (Osaka, Japan) and The Institute of Medical Science, The University of Tokyo (Tokyo, Japan).

### Reagents

Herbal medicine extracts as crude drugs were provided from the Research Center for Medicinal Plant Resources of the National Institutes of Biomedical Innovation, Health and Nutrition, Ibaraki, Japan. Ovalbumin (OVA) protein (Kanto Chemical, Osaka, Japan) and poly(I:C) (synthesized; purity: ≥99%; Sigma-Aldrich, MO), MPLA (synthesized from *E. coli*; MW: 1763.47; InvivoGen, CA), K3-CpG (synthesized (5’-ATC GAC TCT CGA GCG TTC TC-3’); MW: 6349.37; endotoxin level: <0.5 EU/mg; purity: ≥90%; GeneDesign, Osaka, Japan), D35-CpG (synthesized (5’-GGT GCA TCG ATG CAG GGG GG-3’); MW: 6327.33; endotoxin level: <0.5 EU/mg; purity: ≥90%; GeneDesign), K3-SPG prepared as previously described (12), c-di-GMP (synthesized; endotoxin level: <25 EU/g; Yamasa, Chiba, Japan), and 3’,3’-cGAMP (synthesized; endotoxin level: <25 EU/g; Yamasa) were used as an antigen and relatively known control adjuvants, respectively. Among the control adjuvants, MPLA is dissolved in DMSO, and others are dissolved in water. For immunization and *in vitro* stimulation, the herbal medicine extracts and the control adjuvants were adjusted with PBS and RPMI1640 medium, respectively.

### Preparation of Herbal Medicine Extracts

Herbal medicines were pulverized by sample mill SK-MX10 (Kyouritsu Riko, Tokyo, Japan) and extracted with hot water for 2 h. After boiling solid herbal medicines in the water, the remaining solid herbal medicine was removed by using ADVANTEC filter paper (No. 2) (ADVANTEC, Tokyo, Japan), and freeze-dried for 7 d by FDU-1200 (EYELA, Tokyo, Japan). Freeze-dried extracts were reconstituted in PBS at 1 mg/mL and used the supernatants after 10 min incubation as the herbal medicine extracts. The endotoxin levels of certain herbal medicines containing the underground part of plants have been examined by providers and endotoxin was not detected in the methanol extract of them.

### Mice Immunizations

After anesthetization, C57BL/6 mice were subcutaneously (s.c.) immunized in the flank of mice with OVA (10 μg) or OVA and each of 73 kinds of herbal medicine extracts (100 μg) or control adjuvants, poly(I:C) (100 μg), MPLA (10 μg), K3-CpG (10 μg), D35-CpG (10 μg), K3-SPG (10 μg), c-di-GMP (10 μg), and 3’,3’-cGAMP (10 μg) in 100 μl solution, on days 0 and 10. Blood was collected on Day 17 from the orbital vein under anesthesia through a heparinized capillary and plasma was collected by centrifugation and spleen was collected on day 17.

### Measurement of Antigen-Specific IgG Titers

Antigen-specific total IgG, IgG1, and IgG2c in plasmas were determined by ELISA as previously described (10). Briefly, 96 well half-area plates (Corning Inc., NY) were coated with carbonate buffer containing 10 μg/mL of OVA overnight at 4°C. After washing, the plates were incubated with 1% BSA in PBS for 1 h at room temperature. The plates were washed and incubated with diluted plasma for 2 h. Then, the plates were washed, and horseradish peroxidase-conjugated anti-mouse IgG, IgG1, or IgG2c antibody (Southern Biotech, AL) was added to the plates. After 1 h, the plates were washed, and TMB Microwell Peroxidase Substrate System (KPL, MD) was added to the wells. After incubation for 20 min, the reaction was stopped by adding 2 N H_2_SO_4_. Antigen-specific antibody titers were defined by log-linear interpolation of the plasma dilution value corresponding to the cut-off absorbance (OD450 of 0.2). For analyses by machine learning algorithms, the values of the fold change of each herbal medicine extract group and each control adjuvant group against the OVA alone group were used.

### Measurement of OVA-Specific IgE

To measure OVA-specific IgE levels in plasma samples, 96 well half-area plates were coated with 0.5 mg/mL purified rat anti-mouse IgE (BD Biosciences, NJ) in PBS and incubated for 2 h at room temperature. After washing, the plates were incubated with 0.1x Block Ace (KAC, Kyoto, Japan) for 1 h at room temperature. The plates were then incubated with standard or diluted plasma overnight at 4°C. After washing, Ovalbumin HRP (Bio-Rad, CA) was added to the plates. One hour later, TMB Microwell Peroxidase Substrate System (KPL) was added to the wells. After incubation for 20 min, the reaction was stopped by adding 2 N H_2_SO_4_. The absorbance at OD450 nm was measured and the concentration of OVA-specific IgE in plasma was calculated according to the standard. For analyses by machine learning algorithms, the concentrations of IgE of each herbal medicine extract group and each control adjuvant group, from which the concentration of OVA alone group was subtracted, were used.

### Measurement of OVA-Specific T Cell Cytokines

To measure OVA-specific T cell cytokines, splenocytes obtained from immunized mice were stimulated with OVA (2 μg) in RPMI1640 medium containing 1% penicillin/streptomycin and 10% fetal bovine serum for 48 h. Mouse IL-13 and mIFN-γ in the supernatants were measured by DuoSet ELISA kit (R&D Systems, MN). For analyses by machine learning algorithms, the concentrations of cytokines of each herbal medicine extract group and each control adjuvant group, from which the concentration of OVA alone group was subtracted, were used.

### Measurement of Cytokine Productions from Human Peripheral Blood Mononuclear Cells (hPBMCs) and Mouse Splenocytes

Human PBMCs (LONZA, Basel, Switzerland; Cellular Technology Limited (CTL), OH) and mouse splenocytes obtained from naïve C57BL/6 mice were cultured in RPMI1640 medium containing 1% penicillin/streptomycin and 10% fetal bovine serum and stimulated with herbal medicine extracts (20 μg) or adjuvants (2 μg) for 24 h. Human or mouse cytokines (hIL-1β, hIL-2, hIL-4, hIL-5, hIL-6, hIL-7, hIL-8, hIL-10, hIL-12p70, hIL-13, hIL-17, hG-CSF, hGM-CSF, hIFN-γ, hMCP-1, hMIP-1β, hTNF-α, and hIFN-α; mIL-1α, mIL-1β, mIL-2, mIL-3, mIL-4, mIL-5, mIL-6, mIL-9, mIL-10, mIL-12p40, mIL-12p70, mIL-13, mIL-17A, mEotaxin, mG-SCF, mGM-CSF, mIFN-γ, mKC, mMCP-1, mMIP-1α, m MIP-1β, mRANTES, and mTNF-α) in the supernatants were measured using Bio-Plex (Bio-Rad) or ELISA kits (PBL Assay Science, NJ) according to the manufacturer’s instructions. For analyses by machine learning algorithms, the concentrations of cytokines of each herbal medicine extract group and each control adjuvant group, from which the concentration of non-stimulated (medium) group was subtracted, were used.

### Nanoparticle Analysis by BD Influx

Each herbal medicine extract and the control adjuvants (100 μg/mL) placed in a tube was set on a jet-in-air-based BD Influx Cell Sorter equipped with small particle option (BD Biosciences, CA) and analyzed at the same flow rate for 1 min/sample. Nano-sized particles were detected and represented as dot plots on an FSC-SSC gating using a 488-nm laser. The detected nano-sized particles were categorized into 16 populations in FSC and SSC plots, and the particle number of each population in PBS was subtracted from the particle number of each population of the samples, and then which was used for calculating the percentage of each population for analyses.

### Data Integration and Discriminant Analyses

The mixOmics package (65, 66) in R (67) was utilized for data integration and analyses. For regularized canonical correlation analysis (rCCA), we applied the *tune.rcc ()* function for calculating correlation coefficient. The correlation coefficient was presented by the *cim ()* function on heatmap. For sparse-partial least squares (sparse-PLS), after scaling the integrated data by the *scale ()* function, each adjuvanticity *in vivo* data was assigned as objective variables Y and 10 parameters were assigned as the number selected on each block by the *list.keepX ()* function. In addition, the *block.spls ()* and the *plotLoadings ()* function were performed to analyze and project variables on the loading plot. For data integration analysis for biomarker discovery using latent component method for omics studies (DIABLO), after scaling the integrated data by the *scale ()* function, each adjuvanticity *in vivo* data was assigned as objective variables Y and 10 parameters were assigned as the number selected on each block by the *list.keepX ()* function. Moreover, the *tune.block.splsda ()* function with 3 times fivefold validation, the *block.splsda ()* function and the *plotLoadings ()* function were performed to analyze and project variables on the loading plot.

### Principal Component Analysis (PCA)

PCA was performed using the *prcomp ()* function with scaling. Dot plots were described based on the values of components 1 and 2 using GraphPad Prism (GraphPad Software version 7.03).

## Results

### Seventy-Three Herbal Medicine Extracts Show a Variety of Adjuvanticity Properties, with Characteristic Chemokine Induction in Human and Mouse Primary Immune Cells

**Figure 1:**
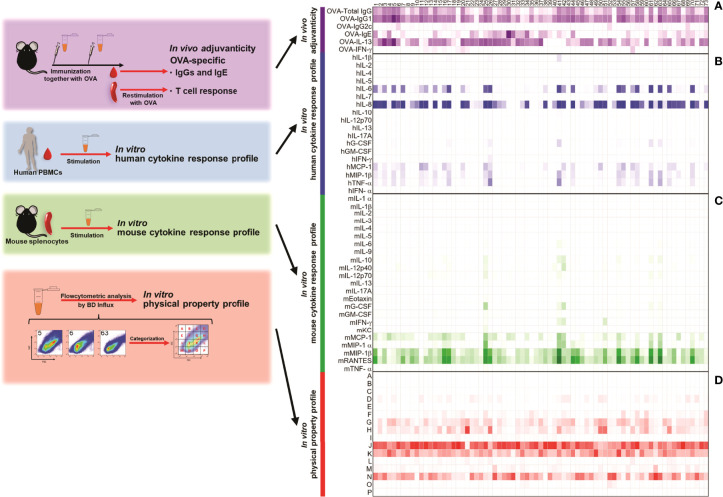
Seventy-three kinds of herbal medicine extracts show a variety of adjuvanticity properties, together with a variety of cytokine inductions in human and mouse primary cells. **(A)** C57BL/6 mice were subcutaneously immunized with 10 μg of OVA and 100 μg of each herbal medicine extract on Days 0 and 10 (n = 3 or 6 mice, each group). On Day 17, plasma and spleen were collected and OVA-specific antibody in plasma and OVA-specific cytokines were measured. Anti-OVA total IgG, IgG1 and IgG2c titers, and IgE level were measured by ELISA. OVA-specific cytokines in the culture supernatants were measured by ELISA after splenocytes were restimulated with OVA for 48 h. Values are shown as the mean (n = 3) in two independent experiments. **(B)** Human PBMCs were stimulated with herbal medicine extract (20 μg) for 24 h and the cytokine levels in the culture supernatants were measured by Bio-Plex or ELISA. Values are shown as the mean of three donors. **(C)** Mouse splenocytes were stimulated with herbal medicine extracts (20 μg) for 24 h and the cytokine levels in the culture supernatants were measured by Bio-Plex. Values are shown as the mean of three independent mice. **(D)** Herbal medicine extracts were analyzed by BD Influx using an FSC-SSC gating. The percentage of each population is shown.

First, we obtained hot water-soluble extracts of 73 herbal medicines, some of which including *Astragali Radix* (#1), *Scutellariae Radix* (#2), *Polygalae Radix* (#6), *Glycyrrhizae Radix* (#8), *Platycodi Radix* (#9), *Ginseng Radix* (#39), *Ophiopogonis Radix* (#40), *Pinelliae Tuber* (#41), *Poria* (#43), and *Ginseng Radix Rubra* (#56) have already been reported to function as adjuvants *in vivo*, although their disease applications are diverse (see *Materials and Methods* and **Table 1** for the details of herbal medicines and preparation of hot-water soluble extracts). To assess their adjuvanticity *in vivo*, mice were subcutaneously immunized twice with a model antigen; chicken ovalbumin (OVA) alone or with each extract as an adjuvant. One week after the second immunization, the plasmas and spleens were collected and, subsequently, OVA-specific antibodies and OVA-specific T cell cytokines by restimulation with OVA were measured. As a result, we found that some extracts induced OVA-specific antibody responses or T cell responses. In detail, #3, #4, #5, #6, #11, #55, and #63 significantly induced OVA-specific total IgG (OVA-Total IgG) compared to OVA alone (**Figure 1A**; **Supplementary Figure 1A**). In terms of IgG subclass, which is one of the most frequent indicators to distinguish the type of adjuvants, Th1-type and Th2-type, which are reflected by IgG2 and IgG1, respectively, #3, #5, #6, #11, #44, #55, and #63 significantly induced higher titers of OVA-IgG1, and #6, #52, and #69 significantly induced a higher titer of OVA-IgG2c than OVA alone (**Figure 1A**; **Supplementary Figures 1B, C**). In addition, #1, #4, #30, #31, #34, and #44 showed higher levels of OVA-specific IgE (OVA-IgE), which is one of the markers for reactogenicity such as induction of allergic immune response, compared to OVA alone (**Figure 1A**; **Supplementary Figure 1D**). In terms of T cell response, #3, #5, #25, and #37 significantly induced higher levels of OVA-specific IL-13 (OVA-IL-13), and #20 and #57 significantly induced OVA-specific IFN-γ (OVA- IFN-γ) compared to OVA alone (**Figure 1A**; **Supplementary Figures 1E, F**). Therefore, the library of the 73 herbal medicine extracts shows heterogeneous adjuvanticity *in vivo* and would be suitable for comprehensive analysis to identify parameters that reflect adjuvanticity *in vivo*.

**Table 1:** List of herbal medicine extracts.

No.	Serial No.	English name	Latin name	Production area	Reports as adjuvant in mice
1	NIB-0267	Astragalus Root	*Astragali Radix*	China	(45, 62)
2	NIB-0001	Scutellaria Root	*Scutellariae Radix*	China	(45)
3	NIB-0250	Phellodendron Bark	*Phellodendri cortex*	Japan	
4	NIB-0094	Coptis Rhizome	*Coptidis Rhizoma*	China	
5	NIB-0185	Coptis Rhizome	*Coptidis Rhizoma*	Japan	
6	NIB-0260	Polygala Root	*Polygalae Radix*	China	(52)
7	NIB-0257	Pueraria Root	*Puerariae Radix*	Korea	
8	NIB-0176	Glycyrrhiza	*Glycyrrhizae Radix*	China	(35, 37)
9	NIB-0258	Platycodon Root	*Platycodi Radix*	China	(42, 55–58, 61, 63)
10	NIB-0272	Apricot Kernel	*Armeniacae Semen*	China	
11	NIB-0222	Cinnamon Bark	*Cinnamomi Cortex*	China	
12	NIB-0248	Magnolia Bark	*Magnoliae Cortex*	Japan	
13	NIB-0120	Achyranthes Root	*Achyranthis Radix*	China	
14	NIB-0423	Euodia Fruit	*Euodiae Fructus*	China	
15	NIB-0246	Schisandra Fruit	*Schisandrae Fruits*	Japan	
16	NIB-0121	Bupleurum Root	*Bupleuri Radix*	China	
17	NIB-0273	Asiasarum Root	*Asiasari Radix*	China	
18	NIB-0020	Gardenia Fruit	*Gardeniae Fructus*	China	
19	NIB-0421	Cornus Fruit	*Corni Fructus*	China	
20	NIB-0274	Japanese Zanthoxylum Peel	*Zanthoxyli Piperiti Pericarpium*	Japan	
21	NIB-0255	Dioscorea Rhizome	*Dioscoreae Rhizoma*	China	
22	NIB-0155	Rehmannia Root	*Rehmanniae Radix*	China	
23	NIB-0156	Rehmannia Root	*Rehmanniae Radix*	China	
24	NIB-0129	Peony Root	*Paeoniae Radix*	Japan	
25	NIB-0047	Plantago Seed	*Plantago Seed*	China	
26	NIB-0110	Ginger	*Zingiberis Rhizoma*	China	
27	NIB-0132	Cnidium Rhizome	*Cnidii Rhizoma*	Japan	
28	NIB-0058	Atractylodes Lancea Rhizome	*Atractylodis Lanceae Rhizoma*	China	
29	NIB-0160	Perilla Herb	*Perillae Herva*	China	
30	NIB-0134	Rhubarb	*Rhei Rhizoma*	China	
31	NIB-0135	Rhubarb	*Rhei Rhizoma*	China	
32	NIB-0420	Jujube	*Zizyphi Fructus*	China	
33	NIB-0264	Alisma Tuber	*Alismatis Tuber*	China	
34	NIB-0259	Anemarrhena Rhizome	*Anemarrhenae Rhizoma*	China	
35	NIB-0262	Uncaria Hook	*Uncariae Uncis Cam Ramulus*	China	
36	NIB-0253	Citrus Unshiu Peel	*Citri Unshiu Pericarpium*	China	
37	NIB-0136	Japanese Angelica Root	*Angelicae Actilobae Radix*	China	
38	NIB-0271	Peach Kernel	*Persicae Semen*	China	
39	NIB-0056	Ginseng	*Ginseng Radix*	China	(46–48)
40	NIB-0418	Ophiopogon Root	*Ophiopogonis Radix*	China	(40)
41	NIB-0430	Pinellia Tuber	*Pinelliae Tuber*	China	(53, 54)
42	NIB-0050	Atractylodes Rhizome	*Atractylodis Rhizoma*	China	
43	NIB-0140	Poria Sclerotium	*Poria*	China	(43)
44	NIB-0403	Sinomenium Stem and Rhizome	*Sinomeni Caulis Et Rhizoma*	Japan	
45	NIB-0244	Saposhnikovia Root and Rhizome	*Saposhinkoviae Radix*	China	
46	NIB-0417	Moutan Bark	*Moutan Cortex*	China	
47	NIB-0141	Ephedra Herb	*Ephedrae Herba*	China	
48	NIB-0252	Akebia Stem	*Akebiae Caulis*	Japan	
49	NIB-0755	Clematis Root	*Clematidis Radix*	China	
50	NIB-0783	Corydalis Tuber	*Corydalis Tuber*	China	
51	NIB-0788	Processed Ginger	*Zingiberis Rhizoma Processum*	China	
52	NIB-0804	Chrysanthemum Flower	*Chrysanthemi Flos*	China	
53	NIB-0833	Immature Orange	*Aurantii Fructus Immaturus*	Japan	
54	NIB-0838	Notopterygium	*Notopterygii Rhizoma*	China	
55	NIB-0854	Schizonepeta Spike	*Schizonepetae Spica*	China	
56	NIB-0863	Red Ginseng	*Ginseng Radix Rubra*	Japan	(47–49, 51, 59)
57	NIB-0873	Jujube Seed	*Zizyphi Semen*	China	
58	NIB-0896	Lithospermum Root	*Lithospermi Radix*	China	
59	NIB-0901	Cimicifuga Rhizome	*Cimicifugae Rhizoma*	China	
60	NIB-0917	Magnolia Flower	*Magnoliae Flos*	China	
61	NIB-0939	Polyporus Sclerotium	*Polyporus*	China	
62	NIB-0962	Gastrodia Tuber	*Gastrodiae Tuber*	China	
63	NIB-0998	Aralia Rhizome	*Araliae Cordatae Rhizoma*	Korea	
64	NIB-1002	Mentha Herb	*Menthae Herba*	China	
65	NIB-1026	Angelica Dahurica Root	*Angelicae Dahuricae Radix*	Korea	
66	NIB-1044	Processed Aconite Root	*Aconiti Radix Processa*	China	
67	NIB-1050	Quercus Bark	*Quercus Cortex*	Japan	
68	NIB-1053	Hemp Fruit	*Cannabis Fructus*	China	
69	NIB-1060	Saussurea Root	*Saussureae Radix*	China	
70	NIB-1070	Leonurus Herb	*Leonuri Herba*	China	
71	NIB-1082	Longan Aril	*Longan Arillus*	China	
72	NIB-1085	Japanese Gentian	*Gentianae Scabrae Radix*	China	
73	NIB-1097	Forsythia Fruit	*Forsythiae Fructus*	China	

Next, we evaluated the cytokine response profiles of the 73 herbal medicine extracts in human peripheral blood mononuclear cells (hPBMCs) and mouse splenocytes as biological properties. To this end, we stimulated them with the 73 herbal medicine extracts and measured the levels of cytokines in the supernatants. hIL-8, hMCP-1, hMIP-1β; mMCP-1, mMIP-1β; and mRANTES were predominantly induced by numerous herbal medicine extracts compared to other cytokines (**Figures 1B, C**; **Supplementary Figures 2**, **3**). The majority of the extracts that induced high levels of OVA-specific total IgG, #3, #5, #6, #11, #55, and #63, but not #4, induced high levels of human pro-inflammatory cytokines and chemokines (**Figures 1A, B**). These results demonstrate that these herbal medicine extracts function as adjuvants in mice and can activate human immune cells *in vitro* as well. Furthermore, certain extracts such as #4 and #11 showed higher cytokine responses in either hPBMCs or mouse splenocytes, but not in both species, probably due to the differential expression of pattern recognition cell receptors among human and mice immune cell types between or due to the differences in the type of immune cells contained in PBMCs and splenocytes (**Figures 1B, C**; **Supplementary Figure 3**). These results using hPBMCs and mouse splenocytes indicated that certain herbal medicine extracts induce a cytokine/chemokine profile which seems to be a reproducible and potentially useful dataset that could potentially predict adjuvanticity *in vivo*.

### All Herbal Medicine Extracts Contain Nanoparticles With Distinct Size Distribution Profile

Particle properties, such as size, of adjuvants are known to be involved in the induction of certain types of immune responses *in vivo* (14, 21–28, 68). Furthermore, it has previously been reported that nanoparticles composed of polysaccharides, such as arabinogalactan and cellulose, detected in a hot water extract of *Glycyrrhizae Radix*, which is #8 in this study, showed an immunostimulant effect *in vitro* (64). These results led us to hypothesize that the size of adjuvants could be a parameter to estimate the adjuvanticity *in vivo* of candidate adjuvants. Therefore, we attempted to investigate whether herbal medicine extracts contained nanoparticles, which is consistent with a previous study (64).

Generally, transmission electron microscopy or dynamic light scattering are used to analyze the properties of nanoparticles (64). However, it is hard to quantitate each nanoparticle in the samples, and time consuming to analyze a vast number of samples with these techniques. Therefore, we took advantage of a high-resolution flow cytometer (BD Influx) in anticipation of utilizing the size of adjuvant in a screening method. It has already been reported that the BD Influx is suitable for both qualitative and quantitative analysis of nano-sized cell-derived vesicles as small as 100 nm on the basis of light scatters (69), and with significantly less operation time to analyze samples. Next, we analyzed the nanoparticles contained in the hot water extracts of the 73 herbal medicines. Interestingly, we identified several nano-sized particles in soluble extracts of all herbal medicines and their sizes were approximately a few hundred nanometers based on the size of the control beads (**Supplementary Figure 4A**). For further analysis, we categorized particles into 16 populations, populations A to P, based on the mean intensities of FSC and SSC, reflecting their mean diameter and density, respectively (**Figure 1D**; **Supplementary Figure 4B**). Major populations were observed in populations J, K, N, and H (**Figure 1D**). Moreover, there were variations in particle size and density among the extracts (**Figure 1D**, **Supplementary Figure 4A**). This might have been caused by the different components and heterogeneity of herbal medicine extracts. Taken together, these data indicate that the pattern of size and density of nano-sized particles was unique to each herbal medicine extract and might be useful for characterizing the adjuvant properties of herbal medicine extracts.

### Certain Cytokines and Chemokines Produced by Adjuvant-Stimulated Human and Mouse Cells *In Vitro* Are Positively Correlated With the Adjuvanticity *In Vivo* in Mice

**Figure 2:**
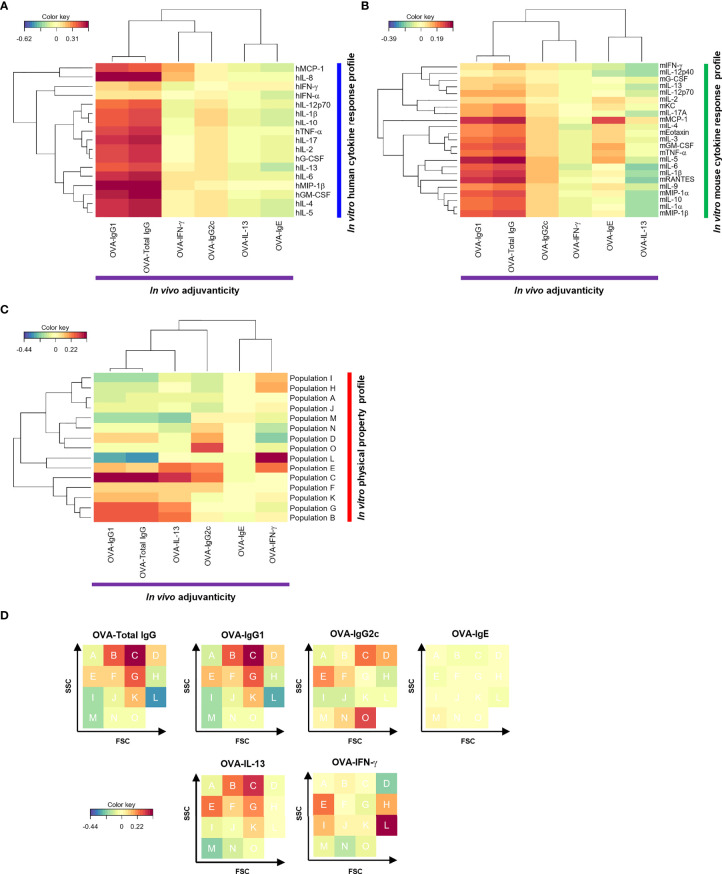
The cytokine response profile and physical property profile of the herbal medicine extracts showed correlation with their adjuvanticity in rCCA. **(A−C)** Correlations between the adjuvanticity *in vivo* and the *in vitro* human cytokine response profile **(A)**, the *in vitro* mouse cytokine response profile **(B)**, or the physical property profile **(C)** of the herbal medicine extracts were calculated by rCCA and they are represented on heatmaps. **(D)** Heatmap of rCCA shown in **(C)** is represented on an FSC-SSC gating.

To identify parameters of adjuvant candidates to predict adjuvanticity *in vivo* by human and mouse cytokine response profiling *in vitro*, we first performed the computational analysis called regularized canonical correlation analysis (rCCA). CCA is a method to extract the correlation between two datasets, and rCCA is its extension to handle high-dimensional datasets with n < p (70). The rCCA between the human cytokines and the adjuvanticity *in vivo* revealed that most of the human cytokines, such as hIL-8, hMIP-1β; and hGM-CSF, were highly positively correlated with OVA-specific IgG1 and total IgG titers of immunized mouse plasmas (**Figure 2A**). In contrast, there were very few human cytokines that were highly correlated with OVA-specific IgG2c, IgE, or T cell cytokines, except for which hMCP-1 and hIL-8 were positively correlated with OVA-IFN-γ while hIL-1β and hIL-10 were positively correlated with OVA-IgG2c (**Figure 2A**). In terms of OVA-specific IL-13 and IgE, their correlation coefficient patterns were similar, and most of the human cytokines were negatively correlated with them (**Figure 2A**). These results demonstrate that the human cytokines showed clear correlations with mouse adjuvanticity *in vivo* characterized by OVA-specific total IgG and IgG1, but not with IgG2c, suggesting the characteristics of the herbal medicine extracts mainly inducing type-2 humoral responses may be predicted by human cytokine responses to them.

We next calculated the correlations between the mouse cytokine responses *in vitro* and the adjuvanticity *in vivo* elicited by the 73 herbal medicine extracts. Mouse IL-5, mMCP-1, and mRANTES showed high correlation with OVA-specific IgG1 (**Figures 1**, **2B**; **Supplementary Figure 3**). Alternatively, few mouse cytokines like mMCP-1 and mIL-5 were highly correlated with other adjuvanticity parameters such as OVA-specific IgE as well to co-administered OVA (**Figures 2A, B**). These data suggest that mouse cytokine profiling using spleen cells *in vitro* may be able to predict the adjuvanticity *in vivo.*

### Correlation Analysis Between Nanoparticle Size/Density Within the Solution and the Adjuvanticity of the 73 Herbal Medicine Extracts Is Possible, Which May Predict the Type of Immune Responses

Next, we examined the correlation between the adjuvanticity *in vivo* and the size and density of nanoparticles found in the herbal medicine extracts by using flowcytometric and rCCA analysis. Flowcytometric measurement of fine particles approximately 100-300 nm in diameter was conducted for all 73 herbal medicine extracts. Nano-sized particles were visualized in 4-digit, log scale dot blots with FSC and SSC divided into 16 populations that are analyzed for correlation with the adjuvanticity (**Supplementary Figure 4**; **Figures 2C, D**). **Figure 2D** shows the same heatmaps as **Figure 2C** in a different way (shown as FSC-SSC FACS plots). To our surprise, the overall correlation coefficient of the physical property profile with the adjuvanticity *in vivo* was higher than that with the human and mouse cytokine response profiles (**Supplementary Figure 5**). The nanoparticles found in the herbal medicine extracts are solubilized in the solution, yet a variety of nanoparticles are quantitated at distinct size and density and revealed that most relevant nanoparticles for type-1 adjuvants (biased toward OVA-IgG2c and OVA-IFN-γ), type-2 adjuvants (biased toward OVA-IgG1 and OVA-IL-13), or others (OVA-IgE) are at different size and density (**Figures 2C, D**). These results indicate that the size and density of nano-sized particles in the solution of herbal medicine extracts may well be correlated with the type of immune responses to OVA antigen, in other words, direction of the adjuvanticity.

### Additional Machine Learning-Assisted Screening Methods Revealed That Certain Human and Mouse Chemokines and Size/Density Profiling *In Vitro* Further Improve to Predict the Adjuvanticity *In Vivo*

Although rCCA allowed us to identify the correlations, it did not indicate that the parameters elucidated in the analysis were effective for screening adjuvants. Therefore, we performed a further machine learning analysis, sparse-PLS, which is a method for simultaneous dimension reduction and feature selection that can handle multicollinearity (71, 72), while rCCA cannot perform variable selection. Here, we attempted to improve to screen and predict adjuvanticity *in vivo* using the human and mouse cytokine response profiles, and the size/density profile by sparse-PLS analysis. Among human and mouse cytokines, chemokines, and growth factors 10 most contributing cytokines showed a positive relativity with OVA-specific IgG1 and total IgG (**Supplementary Figure 6**). In terms of human cytokines, hGM-CSF and hMIP-1β, which showed the highest correlations with OVA-specific IgG1 and total IgG in rCCA, were identified as the top two human cytokines that can best describe the OVA-specific IgG1 and total IgG-inducing adjuvants by sparse-PLS analysis, although hIL-8, which also showed high positive correlations with them, was not identified by sparse-PLS analysis (**Figure 2A**; **Supplementary Figure 6**). This was likely because hIL-8 levels were high even in the extracts with weaker adjuvanticity *in vivo* and appeared to be unsuitable for distinguishing the strength of adjuvanticity *in vivo*. Similar to hGM-CSF and hMIP-1β, sparse-PLS analysis confirmed that mRANTES and mMIP-1β are potential parameters capable of distinguishing OVA-specific IgG1- and total IgG-inducing adjuvants (**Supplementary Figure 6**). In both human and mouse cytokine response profiles, inflammatory cytokines such as IL-6 and TNF-α were surprisingly not determined as the top parameters although they are ranked in the top 10 and they are well-known cytokines for screening and evaluation of adjuvanticity in previous studies. Rather, chemokines and other certain cytokines appeared to be more suitable parameters for predicting adjuvanticity *in vivo* (**Supplementary Figure 6**). In terms of the physical property profile, by sparse-PLS analysis, FSC^high^ G, C, and K populations which showed a positive correlation with OVA-specific IgG1 and total IgG in rCCA, were also elicited as parameters that can distinguish adjuvants with a high ability to induce OVA-specific IgG1 and total IgG responses (**Figures 2C, D**; **Supplementary Figure 6**). Uniquely to sparse-PLS analysis, certain populations, such as M and I (FSC^low^ SSC^low^), showed a negative relativity with OVA-specific IgG1 and total IgG, suggesting that herbal medicine extracts with a high percentage of these populations have a capacity to induce weak OVA-specific IgG1 and total IgG responses *in vivo*. Therefore, sparse-PLS analysis further convince that nanoparticle profiles of the candidate adjuvants could be used to predict their adjuvant activities (**Supplementary Figure 6**), while OVA-specific IgG2c and IgE responses had no cytokines with which showed a positive relativity, distinct from those analyzed by rCCA indicating its limitation (**Figures 2A, B**; **Supplementary Figure 6**).

**Figure 3:**
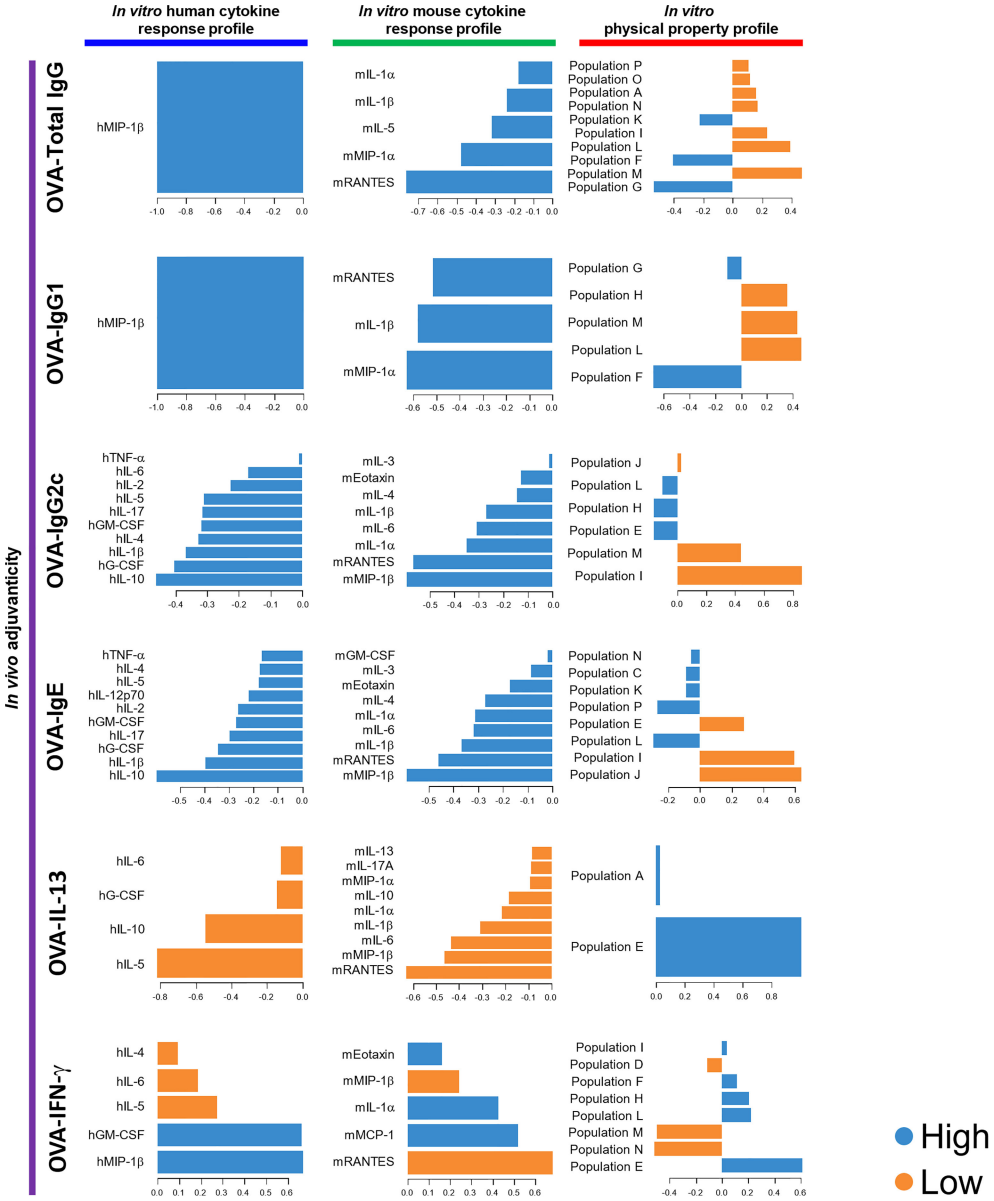
DIABLO using the data of the 73 herbal medicine extracts identified parameters that mostly discriminated the level of each adjuvanticity *in vivo*. Each parameter selected on the first component is represented on loading plots. The parameters indicated by blue and orange are predominantly observed in the high and low group of each adjuvanticity *in vivo*, respectively.

Next, we utilized DIABLO. DIABLO is a function implemented in the mixOmics R package, which is an extension of sparse generalized CCA for discriminant analysis to identify variables that explain the outcome by multiple data integration (73). DIABLO analyzes data in a similar manner to screening, which discriminates potential candidates from unlikely candidates. In this study, the herbal medicine extracts were categorized into two groups, high and low, based on higher or lower levels of adjuvanticity *in vivo* than the average of them, and parameters that can distinguish the high or low groups of the herbal medicine extracts were computed from the human cytokine response, mouse cytokine response, and physical property profiles of 73 herbal medicine extracts. Although not all human and mouse cytokines elicited in DIABLO were consistent with those picked by rCCA or sparse-PLS analysis, hMIP-1β and mRANTES were consistently elicited as the top cytokines in the cytokine response profiles (**Figure 3**, **Supplementary Figure 6**). Furthermore, the flowcytometric physical property profile by DIABLO showed that populations G and F were analyzed as parameters that were dominantly observed in the high group and can distinguish the high levels of OVA-Total IgG and IgG1, while populations M and L were analyzed as negative parameters dominantly observed in the low group, most of which are similar to those obtained by sparse-PLS (**Figure 3**, **Supplementary Figure 6**). These results indicated that the parameters elicited by three machine learning algorithms rCCA, sparse-PLS, and DIABLO, which are highly related to adjuvanticity *in vivo*, would be useful for adjuvant screening, although the accuracy and specificity may vary between each algorism.

### Validation Study Using the Known Adjuvants Confirmed the Utility of the Three Machine Learning-Assisted Screening of Adjuvants

**Figure 4:**
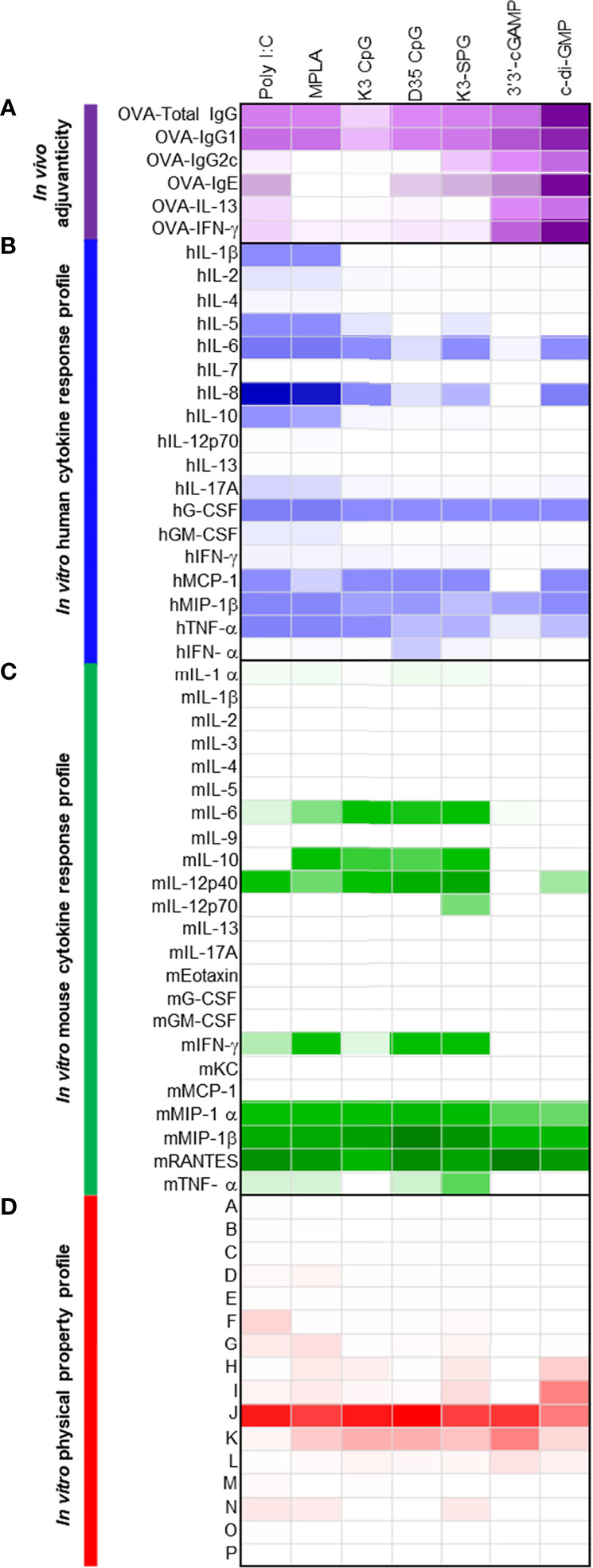
Seven control adjuvants show a variety of adjuvanticity properties, together with a variety of cytokine inductions in human and mouse primary cells. **(A)** C57BL/6 mice were subcutaneously immunized with 10 μg of OVA and 10 or 100 μg of each control adjuvant on Days 0 and 10 (n = 5 mice, each group). On Day 17, plasma and spleen were collected and OVA-specific antibody in plasma and OVA-specific cytokines were measured. Anti-OVA total IgG, IgG1 and IgG2c titers, and IgE levels were measured by ELISA. OVA-specific cytokines in the culture supernatants were measured by ELISA after splenocytes were restimulated with OVA for 48 h. Values are shown as the mean (n = 5). **(B)** Human PBMCs were stimulated with control adjuvants (2 μg) for 24 h and the cytokine levels in the culture supernatants were measured by Bio-Plex or ELISA. Values are shown as the mean of three donors. **(C)** Mouse splenocytes were stimulated with control adjuvants (2 μg) for 24 h and the cytokine levels in the culture supernatants were measured by Bio-Plex. Values are shown as the mean of three independent mice. **(D)** Herbal medicine extracts were analyzed by BD Influx using an FSC-SSC gating. The percentage of each population is shown.

We next determined if the parameters elicited by sparse-PLS and DIABLO, based on the data of herbal medicine extracts, were broadly useful for other soluble adjuvants or limited to herbal medicine extracts. We, therefore, set up a validation study and evaluated the adjuvanticity *in vivo*, human and mouse cytokine response profiles, and physical properties of seven known adjuvants, poly(I:C), MPLA, K3 CpG, D35 CpG, K3-SPG, 3’3’-cGAMP, and c-di-GMP, which or its derivatives are relatively broadly used in preclinical mouse studies or clinically approved or applied in clinical trials, as control adjuvants. The human cytokine responses of PBMC, mouse cytokine responses of spleen cells as well as their physical property were assessed *in vitro* that are compared to their adjuvanticity assessed *in vivo* (**Figures 4A–D**; **Supplementary Figures 7**–**10**).

As the seven known adjuvants showed potent adjuvanticity in both B cell and T cell responses to immunized antigen OVA consistent with previous results, the heatmaps generated by human and mouse cytokines, chemokines, and growth factors *in vitro* were distinct from those with 73 herbal medicines, as the most of the known seven adjuvants are potent immunostimulants in the innate immune system, including agonists for TLRs and RLRs (**Figures 4A–D**).

**Figure 5:**
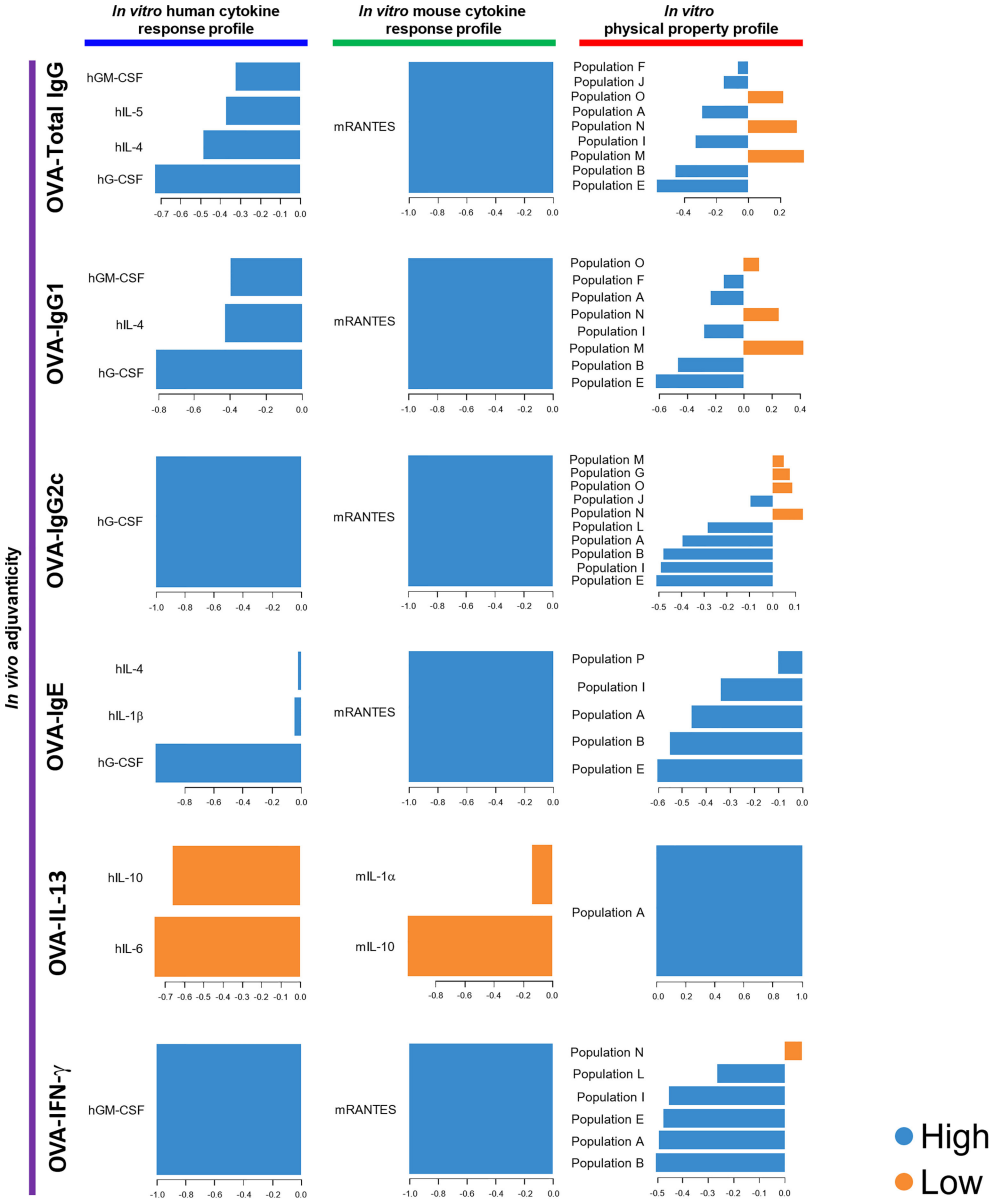
DIABLO using the data of the 73 herbal medicine extracts and the seven control adjuvants identified parameters that mostly discriminated the level of each adjuvanticity *in vivo*. Each parameter selected on the first component are represented on loading plots. The parameters indicated by blue and orange are predominantly observed in the high and low group of each adjuvanticity *in vivo*, respectively.

In order to validate the results obtained by 73 herbal medicines for the three machine learning-assisted adjuvant screening, we then combined the data of the adjuvanticity *in vivo*, cytokine response profiles, and physical property profile of both the 73 herbal medicine extracts and the seven known adjuvants, and performed DIABLO to identify parameters with the highest potential, to discriminate the adjuvanticity *in vivo* of those elicited by the herbal medicine from those elicited by the seven known adjuvants. The results from the combined data sets revealed that hG-CSF was identified in all the blocks, except for OVA-IL-13; ranking first in the human cytokine response profile, while hMIP-1β was the best for the screening of herbal extracts (**Figures 3**, **5**). For mouse cytokines, only mRANTES showed the highest potential in distinguishing the high groups in terms of the levels of OVA-Total IgG, OVA-IgG1, OVA-IgG2c, OVA-IgE, and OVA-IFN-γ (**Figure 5**). Taken together, hG-CSF and mRANTES were the most suitable cytokines for the identification of candidates that can induce high levels of a variety of adjuvanticity (**Figures 3**, **5**).

By using flowcytometric physical property analysis, we happened to detect distinct nanoparticles in all the seven known ‘soluble’ adjuvants (**Figure 4D**; **Supplementary Figure 10**). The population patterns of particles, based on their size and density, were different among the control adjuvants; however, K3 CpG, D35 CpG, and 3’3’-cGAMP showed a similar population pattern with a broad FSC in FSC and SSC plot (**Figure 4D**; **Supplementary Figure 10**). The most abundant population was population J, which was almost consistent with that of the herbal medicine extracts (**Figures 1D**, **4D**).

Yet, there was not a strong correlation of those populations with adjuvanticity *in vivo* except population M being a negative parameter for OVA-Total IgG, OVA-IgG1, consistent with those obtained by 73 herbal medicines alone.

### Three Machine Learning-Assisted Screening of Adjuvants Are Further Strengthened by PCA Discrimination Using Promising Parameters

**Figure 6:**
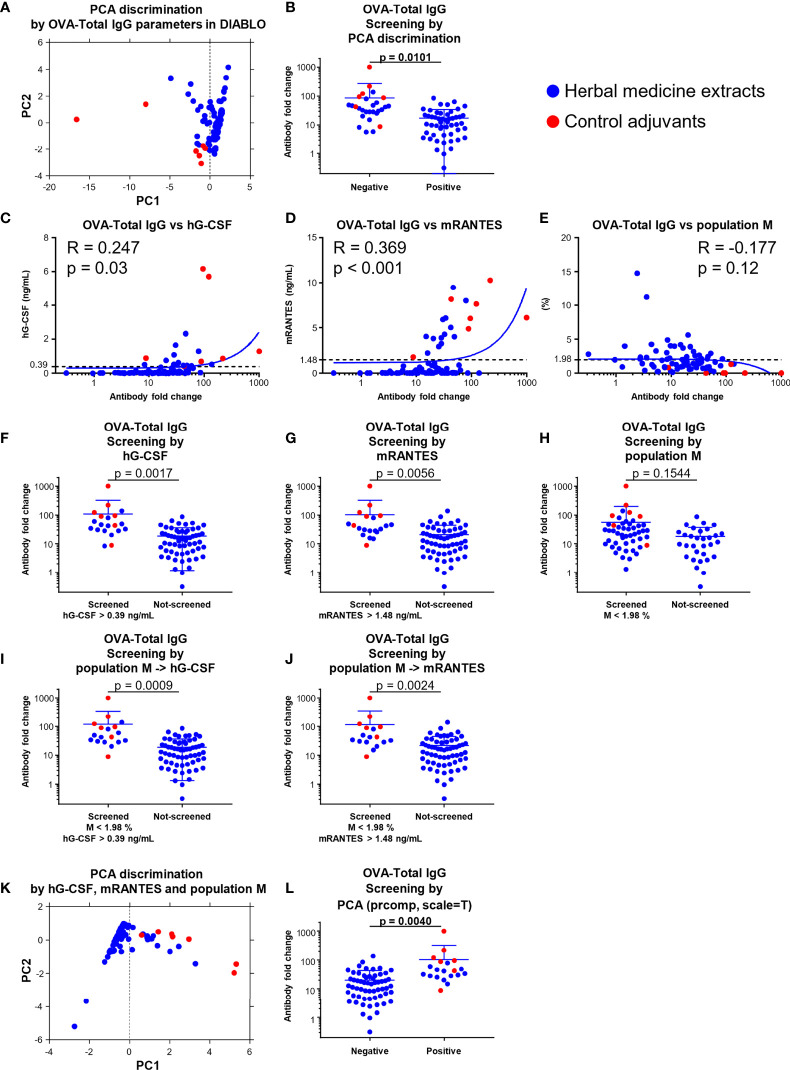
A combination of hG-CSF/mRANTES and population M, and PCA discrimination; using them improved screening efficiency. **(A)** PCA discrimination on the data of the 73 herbal medicine extracts and the seven control adjuvants calculated by the levels of the parameters identified in the OVA-Total IgG block by DIABLO were performed. **(B)** The fold change of OVA-Total IgG against OVA alone of the 73 herbal medicine extracts and the seven control adjuvants grouped into positive and negative based on PCA discrimination shown in **(A)** at the threshold of the average of component 1. (Unpaired t test). **(C−E)** Correlation between fold change level of OVA-Total IgG against OVA alone and the values of hG-CSF **(C)**, mRANTES **(D)**, or population M **(E)**. **(F−J)** Theoretical screenings of the 73 herbal medicine extracts and the seven adjuvants using the levels of hG-CSF, mRANTES, and population M (**F**; hG-CSF, **G**; mRANTES, **H**; population M, **I**; population M and hG-CSF, **J**; population M and mRANTES). (Unpaired t test). **(K)** PCA discrimination on the data of the 73 herbal medicine extracts and the seven control adjuvants calculated by the levels of hG-CSF, mRANTES, and population M. **(L)** The fold change of OVA-Total IgG against OVA alone of the 73 herbal medicine extracts and the seven control adjuvants grouped into positive and negative based on PCA discrimination shown in (**K**) at the threshold of the average of component 1. (Unpaired t test).

Finally, in order to further improve the potential of machine learning-assisted screening of adjuvants as shown above, we attempted to use PCA, which reduces the dimensionality while retaining the variance in the data as much as possible and summarizing the data in a scatter plot to confirm the quality of the identified parameters, based on the values of the parameters identified in the OVA-Total IgG block of DIABLO (**Figure 6A**; **Supplemental Table 1**). The seven known ‘control’ adjuvants and some herbal medicine extracts were differentiated from other herbal medicine extracts in component1, and we therefore evaluated the titer of OVA-Total IgG of component1 negative group including the control adjuvants and component1 positive group (**Figures 6A, B**). By this evaluation, we could successfully distinguish the control adjuvants and some herbal medicine extracts with high OVA-specific total IgG-inducing potential, in particular in combination with DIABLO, not sparse-PLS (**Figure 6B**; **Supplementary Figure 11**). Consistently, the parameters identified in the blocks of OVA-IgG1 and OVA-IFN-γ could identify the control adjuvants and some herbal medicine extracts with high OVA-specific IgG1- or IFN-γ-inducing ability (**Supplementary Figure 12**).

To determine the quality of each parameter identified, we further evaluated the correlation coefficient and p-value of each parameter compared to those of OVA-Total IgG and showed that hG-CSF and mRANTES are the most suitable parameters among human and mouse cytokines identified by DIABLO (**Figures 6C, D**; **Supplementary Figures 13A, B**). Population M, which was identified as a negative parameter for OVA-Total IgG, IgG1, and IgG2c by DIABLO (**Figure 5**), had no significant correlation with OVA-Total IgG (**Figure 6E**).

Moreover, we performed a theoretical screening of the 73 herbal medicine extracts and the seven control adjuvants using the top positive and negative parameters commonly elicited in several blocks of DIABLO and calculated the degree of separation (p-value) between screened and non-screened groups. When we screened the substances by the level of hG-CSF or mRANTES at a threshold of 0.39 ng/ml or 1.48 ng/ml, respectively, which are average concentrations in the 73 herbal medicine extracts and the seven control adjuvants, the p-value was 0.0017 or 0.0056, suggesting that the herbal medicine extracts with higher titer of OVA-Total IgG and the known control adjuvants were screened significantly efficiently by the levels of hG-CSF and mRANTES they induced *in vitro* (**Figures 6F, G**).

In terms of population M, there was no significant difference between the screened and non-screened groups based on 1.98% of population M, which is the average percentage of the 73 herbal medicine extracts and the seven control adjuvants; however, the screened substances showed higher OVA-Total IgG fold change (**Figure 6H**). Moreover, after screening for the first time based on the percentage of population M at 1.98% and re-screening based on the level of hG-CSF at 0.39 ng/ml or mRANTES at 1.48 ng/ml, the degree of separation and p-value become better as 0.0009 and 0.0024, respectively, and these p-values improved from those of the screening by hG-CSF or mRANTES alone (**Figures 6I, J**). In addition, in terms of other adjuvanticity *in vivo*, except for OVA-IgE, the candidates were significantly screened by the two-step system using population M and hG-CSF, and the screening efficiency improved by adding the negative screening step by population M, compared with the single-step screening by hG-CSF (**Supplementary Figures 13C–L**). However, the other parameter candidates, hIL-4, hIL-5, and hGM-CSF, elicited by DIABLO did not show significant differences in silico screenings (**Supplementary Figures 12M–O**). In addition, hIL-6 and mIL-6, which are well-known parameters in conventional adjuvant screenings, also failed to significantly screen the substances, suggesting that IL-6 was not an effective parameter (**Supplementary Figures 13P, Q**). Taken together, population M and hG-CSF/mRANTES were the best negative and positive parameters, respectively, and their combination improved screening efficiency. This two-step screening system, which is a novel strategy, is highly effective for screening substances with higher adjuvanticity. Moreover, IgE, one of the markers for reactogenicity, is an important end point for developing novel adjuvants as well as markers for immunogenicity. Therefore, we also determined the quality of the parameter candidates that were identified to be related to OVA-IgE in DIABLO (**Figure 5**). First, we checked the correlation of the parameter candidates with the level of OVA-IgE, and hIL-1β, hIL-4, and mRANTES did not significantly correlate with OVA-IgE (**Supplementary Figures 13R–T**). However, population I showed a significantly high correlation, which was influenced by several control adjuvants with a high percentage of population I (**Supplementary Figure 12U**). In addition, all the parameters related to OVA-IgE failed to screen for substances with lower OVA-IgE levels (**Supplementary Figures 13V–Y**). Taken together, no parameter among the human and mouse cytokine response profiles and the physical property profiles was able to identify substances that can induce a high level of IgE to avoid the problem of reactogenicity.

At the end of the quality check for the most suitable parameters, we performed PCA on the data of the herbal medicine extracts and the control adjuvants by using the values of hG-CSF, mRANTES, and population M. Accordingly, the herbal medicine extracts with higher titer of OVA-Total IgG and the control adjuvants were separately plotted from other herbal medicine extracts (**Figure 6K**; **Supplemental Table 2**), suggesting that hG-CSF, mRANTES, and population M were the best screening parameters (**Figure 6L**).

## Discussion

Adjuvants are essential components of vaccines, mostly co-administered with vaccine antigen, or built in the vaccine formulation, to induce potent antigen-specific adaptive immune responses to achieve protective vaccine efficacy. Although aluminum salts (alum) are widely and empirically used in human vaccines, there is a great demand for novel and safe adjuvants screened, identified, and developed in more scientific manners, especially for mechanisms-based and disease-specific applications. Therefore, in this study, we aimed to establish a novel screening strategy for novel and safe adjuvants with reliable and specific parameters obtained *in vitro* that reflect the ideal adjuvanticity *in vivo*. The herbal medicine extracts were used as candidate adjuvants owing to their well-established safety profile. In addition, soluble adjuvants with their simple and well-clarified mechanism of action as direct agonists of pattern recognition receptors, but not particle adjuvants such as alum, were used as control adjuvants because any precipitation was not observed in the herbal medicine extracts and the mechanisms of action of alum and other oil-based adjuvants which are particle adjuvants are complicated and not fully clarified.

We first obtained antigen-specific antibody responses, including IgGs as well as IgE, and antigen-specific T cell cytokine responses induced by the herbal medicine extracts as an *in vivo* measure of adjuvanticity. Of the 73 herbal extracts, we found that seven extracts, *Phellodendri cortex* (#3), *Coptidis Rhizoma* (#5), *Polygalae Radix* (#6), *Cinnamomi Cortex* (#11), *Sinomeni Caulis Et Rhizoma* (#44), *Schizonepetae Spica* (#55), and *Araliae Cordatae Rhizoma* (#63), significantly induced OVA-specific IgG1, and three extracts, *Polygalae Radix* (#6), *Chrysanthemi Flos* (#52), and *Saussureae Radix* (#69), significantly induced OVA-specific IgG2c, compared with OVA alone (**Figure 1A**; **Supplementary Figure 1**). Among them, only extract #6 significantly induced both subclasses of IgG. Extract #6 was a hot water extract of *Polygalae Radix*, and its active component, onjisaponin, acted as an adjuvant for influenza vaccine in an *in vivo* mouse model (36, 50, 52). Our data revealed that some were known, some were newly identified, vaccine adjuvants, but the questions of why and how to screen them without doing animal experiments remain.

We chose three *in vitro* measurements that would predict potential adjuvanticity *in vivo*; human PBMCs or mouse splenocytes cytokine response profiles by, and the physical property profile of, the 73 herbal medicine extracts. They are chosen because the difference in the innate immune systems among species could end up failing due to the wrong choice of animal species of cells used for adjuvant screening. For instance, it has been reported that the structure of STING is different between humans and mice, and DMXAA, a strong STING agonist in mice, does not work in humans (74, 75). In addition, it is also known that TLR9, whose expression is restricted to B cells and dendritic cells in humans, can also be detected in mouse macrophages (76–78). Thus, depending on the target cells or pathways of adjuvant candidates, we cannot accurately predict the adjuvanticity of the candidates in humans using only animal models. Therefore, it is necessary to evaluate the activity of adjuvant candidates in human cells in the pre-clinical stage to develop functional adjuvants in humans. Moreover, *in vitro* and *in vivo* mice studies are beneficial for investigating mode of action, pharmacokinetics, and toxicity of adjuvants. Indeed, mice are the most common models for immune research because of the availability of several immune-related gene-deficient mice and their cost-effectiveness.

Based on above, we picked hPBMCs and mouse splenocytes in this study. Although hPBMCs and mouse splenocytes are fairly distinct in terms of cell components, these cells can easily be obtained and used for immunological studies. In addition, they are primary cells, better than single cell lines to catch any biological and immunological events that occur during the interaction between adjuvant components and cells. Although there are limitations of using those primary cells obtained from individual human and mouse to generalize the results toward a simple conclusion, several interesting outcomes resulted.

First, many herbal medicine extracts were immunostimulatory; some induced human inflammatory cytokines, such as hIL-6 and hTNF-α; and chemokines, such as hIL-8, hMCP-1, hMIP-1β, mMCP-1, mMIP-1β; and mRANTES (**Figures 1B, C**; **Supplementary Figures 2, 3**). These inflammatory cytokines and chemokines are commonly used as indicators of the immunostimulatory activity of adjuvants, often induced by strong agonists for TLRs and STING (17–20).

Second, as expected, quite different cytokine production patterns between hPBMCs and mouse splenocytes were observed; for instance, some extracts induced mMCP-1 and hMCP-1, but not mKC, the homolog of hIL-8, despite the robust induction of hIL-8 (**Figures 1B, C**; **Supplementary Figures 2**, **3**). Similar cytokine production pattern was also observed by the seven ‘known’ control adjuvants, although the cytokines that were not induced by the herbal medicine extracts, such as hG-CSF and mIL-6, were highly induced by them (**Figures 4B, C**; **Supplementary Figures 8**, **9**). In terms of the method of stimulation, we did not transfect the herbal medicine extracts or adjuvants, although the ligands of cytosolic sensors such as 3’3’-cGAMP and c-di-GMP, which are STING agonists, usually show higher stimulatory activity with transfection (79, 80).

Nonetheless, those diverse data sets obtained from human and mouse primary cells stimulated by 73 herbal medicine extracts and seven known control adjuvants were analyzed by three different machine learning methods and reveal for the first time that hG-CSF and mRANTES were broadly useful screening parameters for the herbal medicine extracts, TLR ligands, and STING agonists (**Figures 5**, **6**), suggesting that these cytokines can be novel and useful parameters to screen adjuvant candidates regardless of the type of pattern recognition receptor activated by each adjuvant candidate at initial stages of adjuvant discovery and development. Meanwhile, this study did not reveal responder cells that induced hG-CSF and mRANTES; therefore, there is a limitation to conclude on the most appropriate cells or cell lines for adjuvant screening.

In addition to the differences in immune systems among species, another complicated factor in adjuvant development is the fact that the *in vivo* mode of action of adjuvants is more complicated than their stimulatory effects observed *in vitro*. This is because apart from cytokine production and the activation of immune cells, which are the common screening parameters, other ‘adjuvant’ pathways exist *in vivo*; there are multiple cells including macrophages, dendritic cells, and non-immune cells at the injected site engulf, are activated, or killed by adjuvant. Adjuvant and antigens within or without the antigen presenting cells such as dendritic cells move into the draining lymph node to meet a variety of other immune cells including T and B cells, all of which play roles in the induction of antigen-specific immune responses (81, 82). These make it difficult for us to accurately evaluate the adjuvanticity *in vivo* of adjuvant candidates based only on the immunological properties observed *in vitro*. Therefore, it is also important to explore additional parameters that reflect the adjuvanticity *in vivo* of candidates rather than their *in vitro* activity to overcome the potential inconsistencies between the results of the *in vitro* and *in vivo* assessment of the adjuvanticity and immunological properties of candidates.

To this end, we focused on the physical properties of adjuvant candidates. Among the physical properties, the size of adjuvants is one of the most important factors that influence adjuvanticity *in vivo* although it has been limited to particle adjuvants thus far (14, 21–28, 68). It has been reported that the water-soluble extracts of herbal medicine contained nano-sized particles, which increased IL-6 production through phagocytosis *in vitro* (64). This led us to hypothesize that nano-sized particles in herbal medicine extracts and other soluble adjuvants may have stimulatory activity and the ability to induce immune response *in vivo*. We first focused on the size and density of herbal medicine extracts, which were analyzed by BD Influx as physical properties. We detected the nano-sized particles in all 73 herbal medicine extracts by BD Influx analysis, and the size was approximately 200–500 nm (**Supplementary Figure 4**). We found that the FSC^middle^ SSC^high^ B, C, and G populations positively correlated with antigen-specific total IgG, IgG1, and IL-13, whereas the SSC^low^ M population negatively correlated with the adjuvanticity (**Figures 2C, D**). Moreover, their correlation coefficients were comparable or higher than the correlation coefficients of several populations with *in vitro* cytokine production (**Figures 2C, D** and **Supplementary Figure 5**), suggesting that the nano-sized particles in the herbal medicine extracts preferentially reflected adjuvanticity *in vivo* rather than *in vitro* stimulatory activity. In addition, human cytokines and mouse cytokines were almost differentially clustered by the correlation pattern of population, and human cytokines dominantly showed positive correlations with the FSC^high^ D, H, and L populations; mouse cytokines dominantly showed positive correlations with FSC^low^ E, I, and J populations (**Supplementary Figure 5**). This suggested that the particles of the herbal medicine extracts differentially stimulated hPBMCs and mouse splenocytes, and this effect was related to particle size. Furthermore, we identified that the soluble adjuvants contained nano-sized particles detected by BD Influx (**Supplementary Figure 10**), although their forms were not clear, and we observed that the proportion of nano-sized particles in the FSC^low^ SSC^low^ population in adjuvants may act as a negative parameter predicting candidates without adjuvanticity *in vivo* (**Figures 6I, J**). These results indicate the novel application of the size and density of nano-sized particles in the screening of adjuvant candidates. Although many aspects remain unclear, we believe that the physical property profile of adjuvants need further analysis to reveal the effects of nano-sized particles on immune responses and sorting of nano-sized particles by BD Influx would allow us to obtain further details on nano-sized particles and their adjuvanticity.

Based on the data of the adjuvanticity *in vivo* and *in vitro* profiles, we performed three machine learning algorithms, which are implemented in the mixOmics R package for multiparametric data analysis, to identify parameters that mostly predicted the adjuvanticity *in vivo* of the candidates. The basis of the machine learning techniques we used in this study were PLS and CCA, both of which are popular methods for multivariate analysis. PLS creates latent vectors so that the covariance between the datasets is maximized. CCA conducts linear transformations of two datasets so that the correlation between the datasets obtained by these transformations is maximized. The comparison of PLS and CCA is discussed elsewhere (83). In terms of comparison of the three algorithms in this study, DIABLO seemed to be very suitable for identifying screening parameters owing to its discrimination algorithm, which allowed the identification of highly correlated signatures in data sets. Moreover, the data of the 73 herbal medicine extracts and the seven control adjuvants have less variety of the adjuvanticity *in vivo* (**Figure 1A**), which were insufficient for regression algorithms such as sparse-PLS to predict the level of the adjuvanticity *in vivo*; hence, sparse-PLS was not suitable for the data set of this study (**Supplementary Figures 6**, **11**). In addition, rCCA gave information only about the correlation of parameters but could not elicit the parameters. Therefore, we utilized rCCA only to assess the potential of the *in vitro* profiles as parameters. As observed for hG-CSF, a moderate correlation with adjuvanticity indicated a good parameter, and hIL-8, mMCP-1, and population C, which showed the highest correlations with adjuvanticity, were not identified as parameters in DIABLO (**Figures 3**, **5**). Our results showed that the correlation with adjuvanticity is a necessary condition but not a sufficient condition for screening parameters, indicating that high correlation obtained by rCCA does not mean suitability as a screening parameter.

Finally, we identified and confirmed hG-CSF and mRANTES as the most suitable cytokines for adjuvant screening (**Figure 6**). In addition, we also demonstrated the application of the FSC^low^ SSC^low^ population, population M, as a negative parameter, which paired with the positive parameters in silico screening models, a two-step screening system, using the data of the herbal medicine extracts and the control adjuvants (**Figure 6**). We clearly showed that pairing the negative and positive parameters improved the efficiency and accuracy of screening compared with screening using the positive parameters alone (**Figure 6**). For the PCA method, candidates with higher adjuvanticity *in vivo* can be automatically screened based on the values of hG-CSF, mRANTES, and population M or OVA-Total IgG-related parameters identified by DIABLO, which are hG-CSF, hIL-4, hIL-5, hGM-CSF, mRANTES, and populations E, B, M, I, N, A, J, and F, without setting their threshold (**Figures 6A, B**; **Supplementary Figure 12**). This is because the PCA algorithm maximizes variance and can clarify the difference in patterns for variables, and this makes screening assisted by the PCA algorithm an extremely useful method; however, the PCA algorithm requires a certain number of samples. When we looked at the contribution level of each parameter utilized in the PCA based on the values of hG-CSF, mRANTES, and population M, hG-CSF and mRANTES comparably contributed to the discrimination by PC1 and population M contributed to PC2 as well as PC1, suggesting that the positive and negative parameters independently seemed to work as parameters for the discrimination (**Supplementary Table 1**).

This study revealed that G-CSF from hPBMCs and RANTES from mouse splenocytes are extremely useful positive parameters for screening adjuvant candidates, and the FSC^low^ SSC^low^ population, termed as population M (the smallest size), was a useful negative parameter for the elimination of candidates without adjuvanticity for improved efficacy and accuracy of screening. Furthermore, we demonstrated the application of machine learning algorithms for adjuvant screening and its establishment, namely, DAIBLO and PCA, respectively. This study thus demonstrated a novel two-step screening system, involving the combination of a positive parameter with a negative parameter and PCA discrimination, and effectively screened the herbal medicine extracts and the control adjuvants that have the capacity to induce a variety of antigen-specific immune responses and adjuvanticity *in vivo*.

## Data Availability Statement

The original contributions presented in the study are included in the article/**Supplementary Material**. Further inquiries can be directed to the corresponding author.

## Ethics Statement

The studies involving human participants were reviewed and approved by The Institute of Medical Science, The University of Tokyo (Tokyo, Japan). The animal study was reviewed and approved by National Institutes of Biomedical Innovation, Health and Nutrition (Osaka, Japan) and The Institute of Medical Science, The University of Tokyo (Tokyo, Japan).

## Author Contributions

EK and KJI proposed this project. KH, KK, BT, HN, EK, and KJI. designed the experiments. KH, TH, and KK performed the experiments. KH and NK analyzed the data. HF, HK, and NK kindly provided the herbal medicine extracts. KH, CC, and KJI wrote the manuscript. KJI supervised this work. All authors contributed to the article and approved the submitted version.

## Funding

This work was funded by a grant from Japan Agency for Medical Research and Development (AMED, grant numbers 19ak0101068h0003, 20ak0101068h0004, and 21ak0101068h0005) and Japan Agency for Science and Technology (JST) CREST (grant number JPMJCR18H1).

## Conflict of Interest

The authors declare that the research was conducted in the absence of any commercial or financial relationships that could be construed as a potential conflict of interest.

## Publisher’s Note

All claims expressed in this article are solely those of the authors and do not necessarily represent those of their affiliated organizations, or those of the publisher, the editors and the reviewers. Any product that may be evaluated in this article, or claim that may be made by its manufacturer, is not guaranteed or endorsed by the publisher.

## Supplementary Material

Supplementary Table 1 List of the contribution level of parameter to PC1 and PC2 in PCA. The contribution level of each parameter utilized in **Figure 6A** to PC1 and PC2 is listed.

Supplementary Table 2 List of the contribution level of parameter to PC1 and PC2 in PCA. The contribution level of each parameter utilized in **Figure 6K** to PC1 and PC2 is listed.
