# Supplementary material for: Machine Learning-Assisted Screening of Herbal Medicine Extracts as Vaccine Adjuvants
Source: Front Immunol. 2022 May 19;13:847616. doi: 10.3389/fimmu.2022.847616 (PMC9160479; doi:10.3389/fimmu.2022.847616)
Supplement: Supplementary Table 1 — List of the contribution level of parameter to PC1 and PC2 in PCA. The contribution level of each parameter utilized in Figure 6A to PC1 and PC2 is listed. [file Table_1.docx]

|  | **PC1** | **PC2** |
| --- | --- | --- |
| hG-CSF | 14.21237 | 1.053412 |
| hIL-4 | 11.29361 | 4.579211 |
| hIL-5 | 13.4744 | 3.610856 |
| hGM-CSF | 13.06449 | 4.318659 |
| mRANTES | 7.522006 | 0.089338 |
| Population E | 11.42664 | 0.135433 |
| Population B | 10.81684 | 0.002994 |
| Population M | 1.0843 | 14.57776 |
| Population I | 0.413964 | 5.333446 |
| Population N | 1.800828 | 23.09056 |
| Population A | 9.548278 | 0.000218 |
| Population O | 1.094145 | 19.29249 |
| Population J | 1.19664 | 18.195 |
| Population F | 3.051483 | 5.720612 |

**Supplementary Table 1**

|  | **PC1** | **PC2** |
| --- | --- | --- |
| hG-CSF | 41.86336 | 11.10955 |
| mRANTES | 44.86703 | 3.283894 |
| Population M | 13.26961 | 85.60656 |

**Supplementary Table 2**
