# Supplementary figures and images for: Machine Learning-Assisted Screening of Herbal Medicine Extracts as Vaccine Adjuvants
Source: Front Immunol. 2022 May 19;13:847616. doi: 10.3389/fimmu.2022.847616 (PMC9160479; doi:10.3389/fimmu.2022.847616)

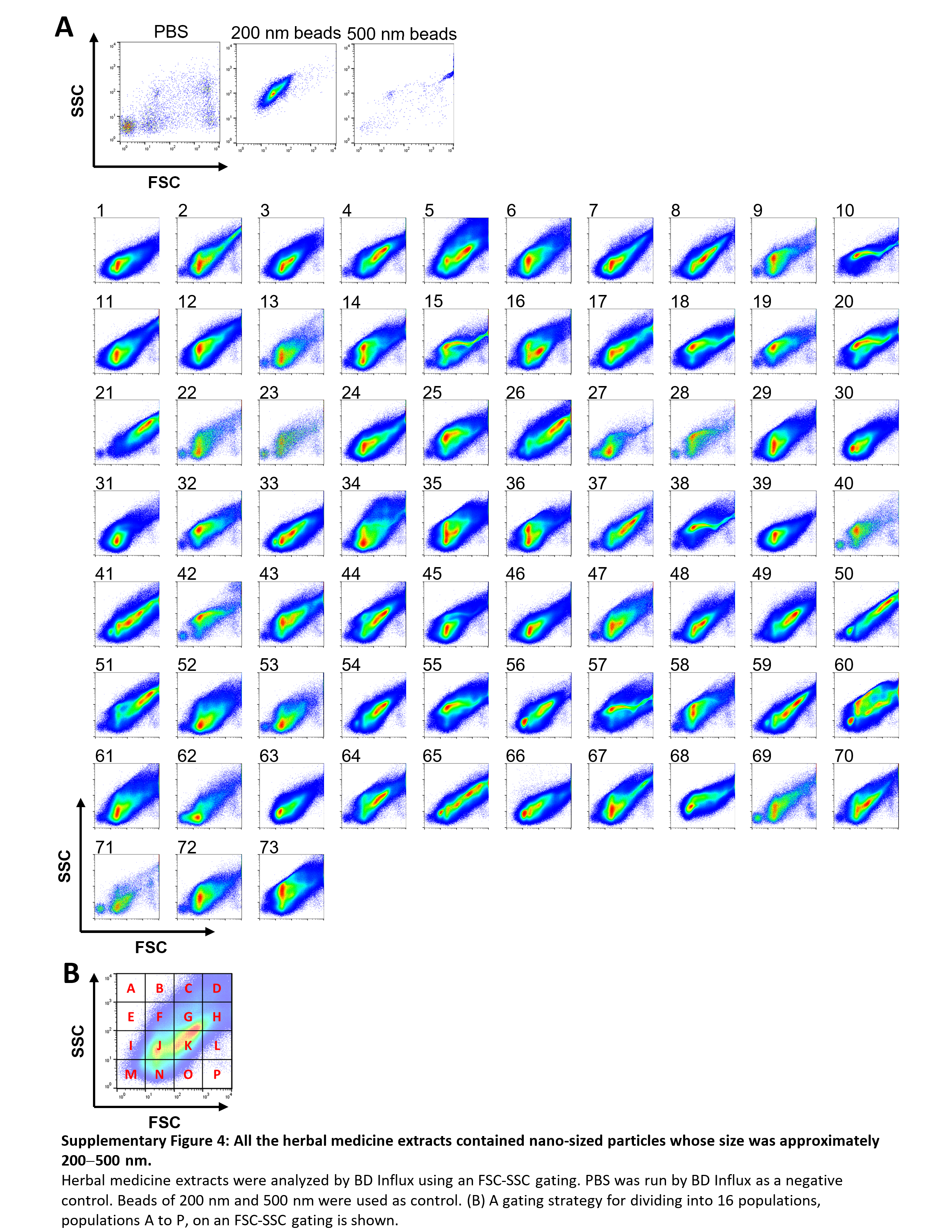

Supplement: Supplementary file 5 [file Image_4.tif]

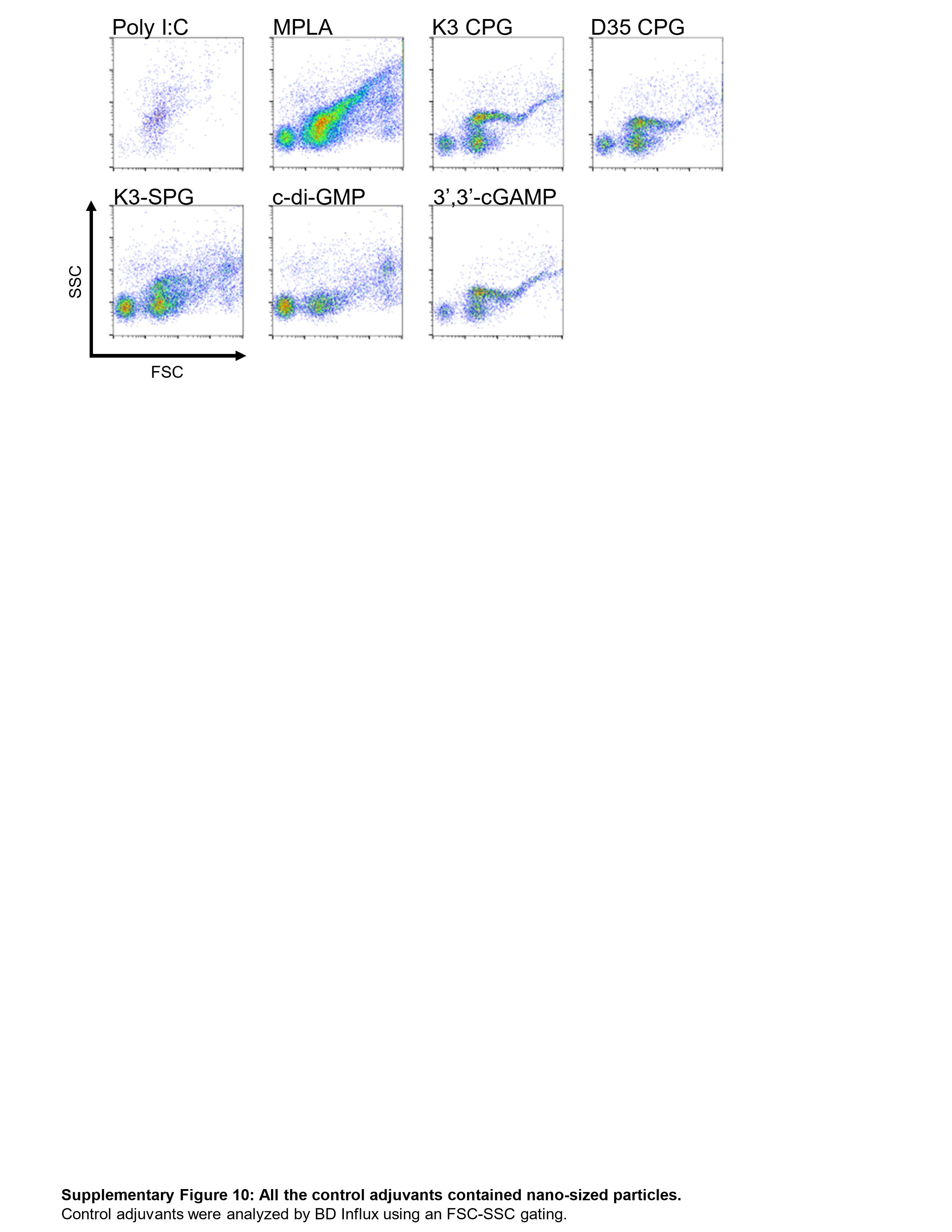

Supplement: Supplementary file 11 [file Image_10.tif]

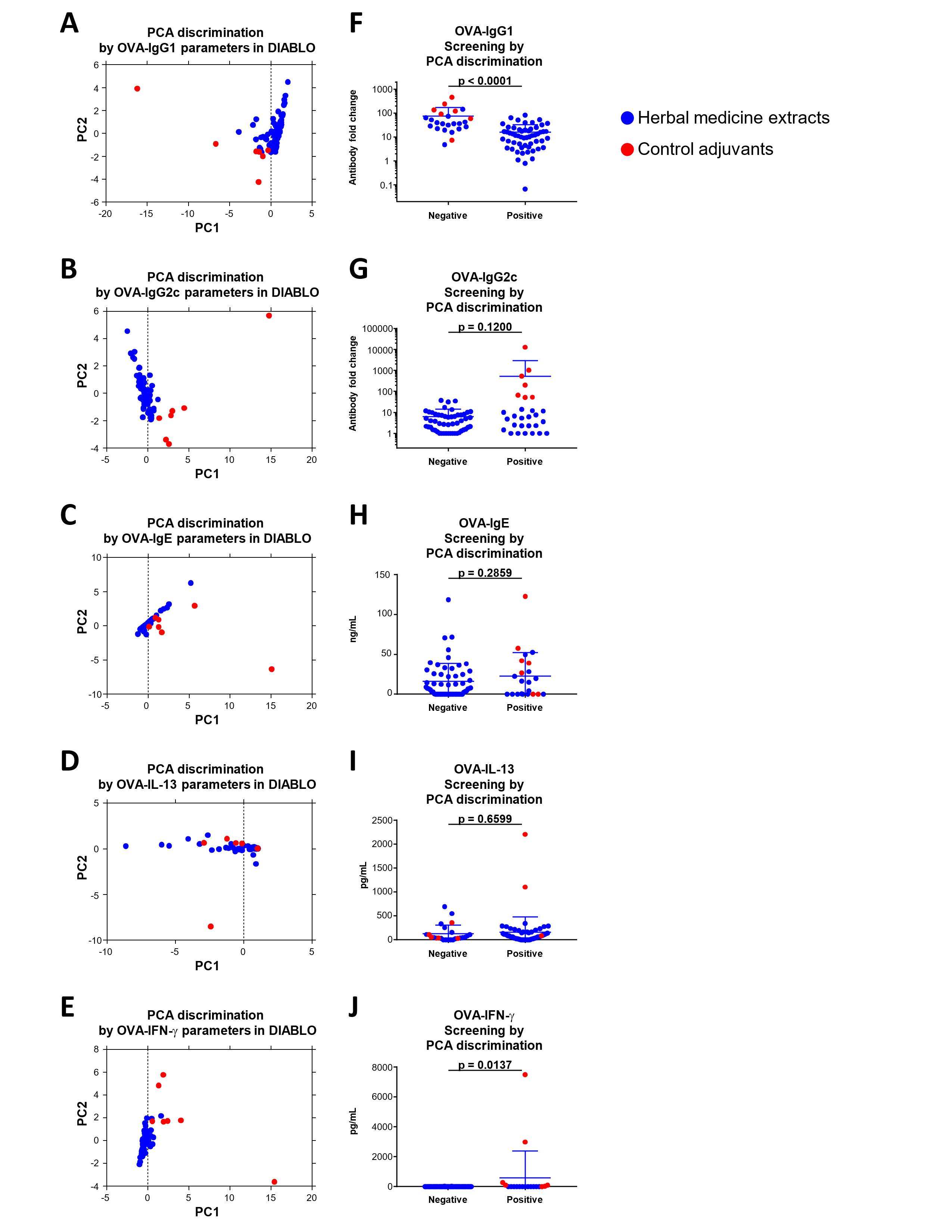

Supplement: Supplementary file 13 [file Image_12.tif]

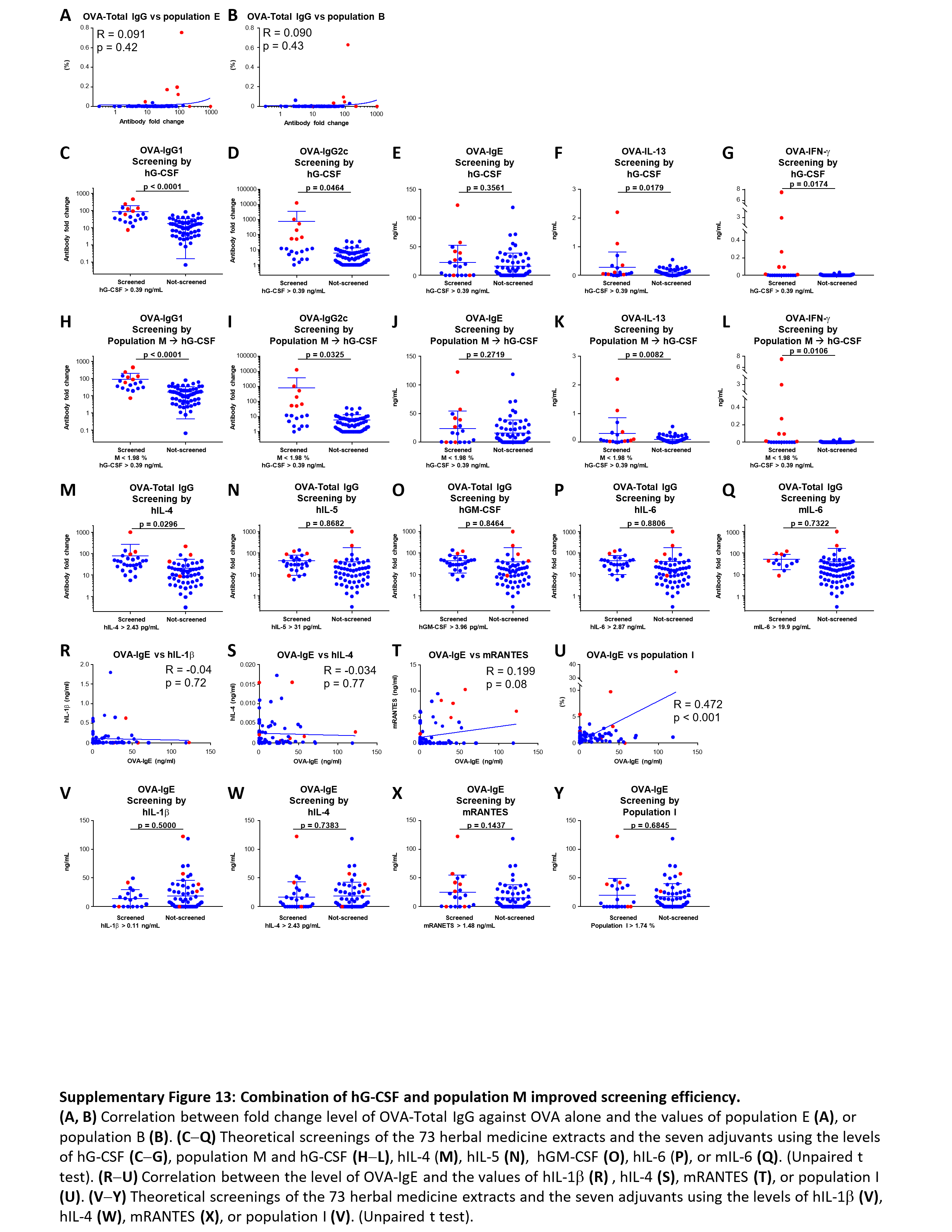

Supplement: Supplementary file 14 [file Image_13.tif]
